# Variations in the use of simple and context-sensitive grapheme-phoneme correspondences in English and German developing readers

**DOI:** 10.1007/s11881-019-00189-3

**Published:** 2020-01-18

**Authors:** Xenia Schmalz, Serje Robidoux, Anne Castles, Eva Marinus

**Affiliations:** 1 Department of Child and Adolescent Psychiatry, Psychosomatics and Psychotherapy, University Hospital, Ludwig-Maximilians-University, Munich, Germany; 2 Department of Cognitive Science, Macquarie University, Sydney, Australia; 3 Macquarie University Centre for Reading (MQCR), Sydney, Australia; 4 Pädagogische Hochschule Schwyz, Goldau, Switzerland

**Keywords:** Context-sensitive rules, Cross-linguistic, Entropy, Grapheme-phoneme correspondences, Reading development

## Abstract

Learning to read in most alphabetic orthographies requires not only the acquisition of simple grapheme-phoneme correspondences (GPCs) but also the acquisition of context-sensitive GPCs, where surrounding letters change a grapheme’s pronunciation. We aimed to explore the use and development of simple GPCs (e.g. a ➔ /æ/) and context-sensitive GPCs (e.g. *[w]a* ➔ /ɔ/, as in “swan” or *a[l][d*] ➔ /o:/, as in “bald”) in pseudoword reading. Across three experiments, English- and German-speaking children in grades 2–4 read aloud pseudowords, where vowel graphemes had different pronunciations according to different contexts (e.g. “hact”, “wact”, “hald”). First, we found that children use context-sensitive GPCs from grade 2 onwards, even when they are not explicitly taught. Second, we used a mathematical optimisation procedure to assess whether children’s vowel responses can be described by assuming that they rely on a mix of simple and context-sensitive GPCs. While the approach works well for German adults (Schmalz et al. in *Journal of Cognitive Psychology, 26*, 831–852, 2014), we found poor model fits for both German- and English-speaking children. Additional analyses using an entropy measure and data from a third experiment showed that children’s pseudoword reading responses are variable and likely affected by random noise. We found a decrease in entropy across grade and reading ability across all conditions in both languages. This suggests that GPC knowledge becomes increasingly refined across grades 2–4.

Most models of single-word reading contain a sublexical route, which converts a string of letters from graphemes to phonemes without relying on whole-word knowledge or semantics (Coltheart, Rastle, Perry, Langdon, & Ziegler, 2001; Perry, Ziegler, & Zorzi, 2007; Perry, Ziegler, & Zorzi, 2010; Plaut, 1999; Plaut, McClelland, Seidenberg, & Patterson, 1996). This phonological decoding process is essential for learning to read (Castles, Rastle, & Nation, 2018; Share, 1995). Decoding a novel word allows children to compute a pronunciation and match it to a word representation which is familiar in its oral form. Thus, phonological decoding provides a means to crack the orthographic code: to start reading in the absence of any word-specific orthographic knowledge. It is therefore important to understand how the knowledge about the print-to-speech correspondences, which drives the phonological decoding process, is acquired.

In the English orthography, some graphemes are pronounced in the same way in most contexts (e.g. *k* ➔ /k/). We refer to such context-insensitive grapheme-phoneme correspondences as simple GPCs. Often, however, the pronunciation of a grapheme is influenced by the context in which it occurs (context-sensitive GPC; hereafter: CS_GPC; Treiman, Kessler, & Bick, 2003; Treiman, Mullennix, Bijeljac-Babic, & Richmond-Welty, 1995; Venezky, 1970): For example, in Australian English (the dialect of the participants in experiments 1 and 3), the *a* in *watt* is pronounced /ɔ/ rather than /æ/, because an *a* preceded by a *w* is often pronounced as in “swan”. Skilled readers show sensitivity to this linguistic regularity: when reading a pseudoword such as *wact*, some participants produce the vowel /æ/ (reflecting sensitivity to a simple GPC), while others produce the vowel /ɔ/ (reflecting sensitivity to a CS_GPC) (Schmalz et al., 2014; Treiman et al., 2003).^1^

By creating pseudowords with specific regularities, one can look beyond readers’ accuracy in reading aloud pseudowords: their transcribed responses provide information about this decoding process (Andrews & Scarratt, 1998; Pritchard, Coltheart, Palethorpe, & Castles, 2012; Schmalz et al., 2014; Steacy et al., 2018; Treiman et al., 2003; Treiman, Kessler, Zevin, Bick, & Davis, 2006). The example of the pseudoword *wact* shows that a given individual’s response can be used to determine whether they relied on a simple GPC or a CS_GPC in that particular instance: we can create pseudowords where different types of GPCs predict different pronunciations. For the pseudoword *hact*, the pronunciation should be unambiguous: The most frequent phoneme that co-occurs with the grapheme *a* is /æ/, and the pronunciation does not change in words where this grapheme is preceded by an *h* (“ham”) or when it is followed by *-ct* (“act”). For the pseudoword *wact*, the *w* preceding the *a* changes its pronunciation to /ɔ/ if the CS_GPC *[w]a* ➔ /ɔ/ is used. This leads to a conflict between the two possible pronunciations, /æ/ and /ɔ/—provided that the reader has picked up on the linguistic regularity that a *w* changes the pronunciation of a subsequent *a* (Treiman et al., 2006).

Often, for monosyllabic words in the English orthography, the CS_GPC involves constraints imposed by the consonants following a vowel rather than the ones preceding it (Treiman et al., 1995). The orthographic unit consisting of the vowel and subsequent consonant(s) of a monosyllabic word is referred to as the body and the pronunciation of this unit as the rime (Duncan, Seymour, & Hill, 2000; Peereman & Content, 1998; Schmalz, Robidoux, Castles, Coltheart, & Marinus, 2017). However, all body-rime correspondences can also be described as CS_GPCs. For example, the vowel *a* is often pronounced as /o:/ when followed by an *l* and another consonant (*a[l][C]* ➔ /o:/, as in “bald” or “salt”). Thus, the pseudoword *hald* has two plausible vowel pronunciations (/æ/ and /o:/), again, depending on whether participants are sensitive to the regularity involving this CS_GPC. Here, the CS_GPC contradicts the simple GPC. We can also create pseudowords where there are two CS_GPCs which contradict the simple GPC, such as *wald*, where the preceding and succeeding consonants suggest the pronunciations /ɔ/ and /o:/, respectively. While we are agnostic about whether there is anything intrinsically different about the processing of CS_GPCs when the modifying grapheme precedes rather than succeeds the modified grapheme, we refer to CS_GPCs which involve the body as CS_GPC_B_, and those involving the onset as CS_GPC_O_, to distinguish between these manipulations in the description of the experimental conditions.

In the current study, we describe data from pseudoword reading aloud experiments with children in grades 2–4: In this age range, children’s reading ability is sufficiently advanced to read aloud pseudowords, but their knowledge of more complex rules is still being shaped by their increasing reading experience (Steacy et al., 2018; Treiman et al., 2006). The design of these experiments is based on our study with English- and German-speaking adults for which we had selected pseudowords where different correspondences (simple GPCs or CS-GPCs) predicted different vowel pronunciations (Schmalz et al., 2014). Based on a linguistic corpus (Baayen, Piepenbrock, & Gulikers, 1995), we calculated the reliability of a given GPC and used this language-level statistic to predict the vowel responses for adult participants. We found that most English and German skilled readers used a mixture of simple GPCs (e.g. *a* ➔ /æ/) and both types of CS_GPCs (*[w]a* ➔ /ɔ/ and *a[l][C]* ➔ /o:/) to pronounce pseudowords. We further hypothesised that any deviation in the participants’ responses from these language-level statistics may reflect individual differences in the extent to which different participants weigh information provided by simple and CS_GPCs. Using an optimisation procedure, we were able to extract weightings from participants’ pseudoword pronunciations, which we proposed as a measure of these individual differences. The current study applied this approach to English-speaking (experiment 1) and German-speaking children (experiment 2). We further seek to extend the analysis to include interactions between types of vowel responses and reading ability and grade in children. We also introduce a new dependent variable, *item-level entropy*, an index of the extent to which different children give different pronunciations to the same grapheme in the same context. Finally, we analyse data from an experiment where the same children read the same pseudowords at different time points, in order to assess the stability of participants’ responses across sessions.

## Experiment 1: modelling vowel responses in English

### Method

#### Participants

Participants were 61 children from a suburban school in New South Wales (Australia). In this school, reading instructions were based on a systematic synthetic phonics approach: CS_GPC_O_ (e.g. *[w]a* ➔ /ɔ/) and CS_GPC_B_ (e.g. *a[l][C]* ➔ /o:/) were not explicitly taught. Twenty-one children were in grade 2, 20 children in grade 3, and 20 in grade 4. The children were tested at the end of the school year. See Table 1 for participant characteristics and their scores on the Sight Word Efficiency subtest of the TOWRE (Torgesen, Wagner, & Rashotte, 1999).

**Table 1:** Participant characteristics in experiment 1: mean (SD)

	Grade 2	Grade 3	Grade 4
Age (months)	96.0 (4.0)	106.6 (6.8)	119.4 (4.0)
TOWRE raw score	55.0 (13.6)	62.7 (9.4)	61.6 (10.8)
TOWRE standardised score	104.2 (17.9)	101.4 (13.5)	90.9 (13.5)

Standardised scores are based on Australian norms (Marinus et al., 2013)

#### Items and procedure

All pseudowords were monosyllabic and contained the vowel grapheme *a*, as its pronunciation in English is heavily dependent on its context. There were four conditions: The pronunciation was either unambiguous (*hact*, the CS_GPC_O_+CS_GPC_B_+ condition, as both the CS_GPC_O_ and the CS_GPC_B_ agree with the simple GPC) or it was changed by one of the context-sensitive rules: By the preceding consonant (*wact*, CS_GPC_O_-CS_GPC_B_+), by the body (*kalt*, CS_GPC_O_+CS_GPC_B_-), or both (*wald*, CS_GPC_O_-CS_GPC_B_-). The items were presented with the software DMDX (Forster & Forster, 2003), in random order, for 4 s or until the voice key was triggered. The recorded responses were transcribed offline by a trained phonologist. The full data and the DMDX script can be found here: https://osf.io/qnuc2/, and a list of items is in Appendix 1.

### Results

The participants’ vowel responses, split up by item Condition and Grade, are presented in Table 2. To explore this pattern of results, we conducted an ANOVA on the number of /æ/ responses across conditions and grades. The 4 × 3 ANOVA included Condition as a within-participant factor and Grade as a between-participant factor. This analysis showed a significant effect of Condition, *F*(3,177) = 258.1, *p* < 0.0001, but no effect of Grade, *F*(1,59) = 0.5, *p* = 0.5, and no interaction between Condition and Grade, *F*(1,177) = 0.3, *p* = 0.9. The effect of Condition shows that participants gave different amounts of /æ/ responses, depending on the context in which the grapheme *a* occurred (see Table 2).

**Table 2:** Summary of responses across conditions in experiment 1; average number of responses (SD)

Condition	Grade	/æ/	/ɔ/	/o:/	Consonant error/ non-response	Other vowel response
CS_GPC_O_+CS_GPC_B_+ (hact)	2	14.6 (2.9)	0.1 (0.3)	0.1 (0.4)	1.5 (2.3)	1.7 (1.6)
	3	16.1 (1.8)	0.1 (0.3)	0 (0)	0.3 (0.6)	1.6 (1.4)
	4	15.8 (2.4)	0.3 (0.6)	0 (0)	0.9 (1.8)	1.2 (1.4)
CS_GPC_O_-CS_GPC_B_+ (wact)	2	9.3 (4.8)	4.3 (4.5)	0.1 (0.4)	1.9 (2.9)	2.4 (2.3)
	3	8.9 (4.0)	5.3 (3.9)	0.2 (0.7)	0.5 (0.6)	3.2 (2.5)
	4	9.9 (4.1)	3.8 (3.9)	0.1 (0.3)	1.0 (1.5)	3.3 (3.1)
CS_GPC_O_+CS_GPC_B_- (kalt)	2	5.9 (3.9)	3.6 (3.6)	1.2 (2.8)	4.9 (4.2)	2.4 (2.8)
	3	4.8 (3.7)	5.8 (4.5)	0.5 (0.9)	2.8 (2.3)	4.1 (4.1)
	4	6.0 (3.9)	5.0 (4.7)	1.1 (3.1)	3.2 (3.5)	2.8 (3.2)
CS_GPC_O_-CS_GPC_B_- (wald)	2	2.1 (3.2)	8.7 (5.7)	0.5 (0.9)	4.3 (4.2)	2.4 (2.7)
	3	1.2 (2.8)	11.2 (4.1)	0.8 (2.2)	2.3 (1.6)	2.6 (3.7)
	4	2.6 (4.6)	10.6 (5.5)	0.8 (2.2)	2.1 (3.3)	2.0 (2.2)

#### Using optimisation to quantify the sensitivity to different GPC types

The optimisation procedure quantifies the extent to which each child relied on simple GPCs, the *[qu/w]a* ➔ /ɔ/−regularity (CS_GPC_O_) and CS_GPC_B_s to compute the pseudowords’ pronunciations. It works by simultaneously fitting three equations for each child and for each of the conditions, with the aim of computing the optimal combination of weightings (*β*_*j*_) for each correspondence *j*, that would describe the probability of this particular child giving a specific response:

$$ P\left(\ae \right)={\beta}_{\mathrm{simple}\ \mathrm{GPC}}\times P\left(\ae |\mathrm{simple}\ \mathrm{GPC}\right)+{\beta}_{{\mathrm{CS}}_{\mathrm{O}}}\times P\left(\ae |{\mathrm{CS}}_O\right)+{\beta}_{{\mathrm{CS}}_{\mathrm{B}}}\times P\left(\ae |{\mathrm{CS}}_{\mathrm{B}}\right) $$

$$ P\left(o:\right)={\beta}_{\mathrm{simple}\ \mathrm{GPC}}\times P\left(o:|\mathrm{simple}\ \mathrm{GPC}\right)+{\beta}_{{\mathrm{CS}}_{\mathrm{O}}}\times P\left(o:|{C\mathrm{S}}_O\right)+{\beta}_{{\mathrm{CS}}_{\mathrm{B}}}\times P\left(o:|{\mathrm{CS}}_{\mathrm{B}}\right) $$

where *P*(phoneme) is the empirically observed percentage of different responses, and *β*_*j*_ denotes the obtained weighting for the use of correspondence *j*, which is multiplied by the predictions from the language corpus (i.e. the conditional probability of a particular phoneme given the probability of this phoneme in all words containing the particular orthographic pattern, *P*(Phoneme|Rule type)). Additional constraints are introduced: namely, that the weights should fall between 0 and 1 (*β*_*j*_ ∈ [0, 1]) and that the sum of the weights should be 1 (∑*β*_*j*_ = 1). This reflects two assumptions: (1) that the weights represent the probability that children rely on a particular correspondence to produce a response, given the item characteristics, and (2) that the three correspondences types are sufficient to predict the participants’ vowel responses (that is, there are no other sources that children can draw on for choosing a response). More detail about the implementation of the optimisation procedure is provided in Schmalz et al. (2014), and the R script can be downloaded here: https://osf.io/cvusr/. Table 3 shows the obtained weights across grades. Before fitting the model, we removed all incorrect responses, three participants (2 from grade 2, 1 from grade 4) with a > 50% error rate and one item (*SLALTZ* in the CS_GPC_O_-CS_GPC_B_- condition) with a > 60% error rate.

**Table 3:** Averaged weights (SD) for the sensitivity to simple GPCs, CS_GPC_O_, and CS_GPC_B_ across Grade in the English sample (experiment 1)

Predictor	Simple GPC	CS_GPC_O_	CS_GPC_B_
Grade 2	0.24 (0.39)	0.63 (0.35)	0.13 (0.14)
Grade 3	0.07 (0.17)	0.79 (0.19)	0.14 (0.13)
Grade 4	0.23 (0.37)	0.63 (0.33)	0.14 (0.15)

To examine whether the model provides an adequate description of the observed data, we calculated the correlation between the observed and model-predicted percentages of different vowel responses. Across grades, these were 0.59 (grade 2), 0.57 (grade 3), and 0.57 (grade 4). Compared to the fits we observed for the adult sample in Schmalz et al. (2014) (0.72 for the English-speaking sample, 0.84 for the German-speaking sample), these model fits are rather poor.

A poor model fit may reflect the use of additional types of GPCs or other decoding strategies, such as guessing, which were not included in the model. One way to test whether the model is simply missing other sources of systematic information is to remove the constraint that the *β* weights should add to 1, as this constraint assumes that the three modeled correspondences (simple GPCs, hereafter sGPC, CS_GPC_O_, and CS_GPC_B_) are sufficient to describe the participants’ responses. If an additional predictor is missing, the model might produce more accurate fits by choosing a set of *β*_s_ that sum to values less than 1. Relaxing this constraint did not substantially change the overall model weights (for grade 2, *β*_sGPC_ = 0.29, *β*_CS_O_ = 0.71, *β*_CS_B_ = 0.16, for grade 3, *β*_sGPC_ = 0.09, *β*_CS_O_ = 0.85, *β*_CS_O_ = 0.17, for grade 4, *β*_sGPC_ = 0.28, *β*_CS_O_ = 0.69, *β*_CS_B_ = 0.17).

#### Item- and participant-level entropy

The above analyses suggest that the modelling approach that was successfully used by Schmalz et al. (2014) for adult readers may not be easily adapted to English-speaking children. This does not seem to be driven by sensitivity to additional types of GPCs. As an alternative explanation, it is possible that the poor model fits reflect a degree of random noise: The children might be less consistent than adults in their vowel responses, regardless of the context in which the vowel occurs. Therefore, we tested if we could quantify individual differences in the extent to which vowel pronunciations may be subject to interference from random noise. To this end, we used *entropy*, a concept from information theory, to examine individual differences in the extent to which vowel pronunciations might be generated by unsystematic, random processes. The entropy measure (*H*) can be used to quantify the diversity of vowel pronunciations (see Borgwaldt, Hellwig, & de Groot, 2004; Coltheart & Ulicheva, 2018, Siegelman, Kearns, & Rueckl, in press for the application of the entropy measure in a similar context). If all participants give the same vowel response to a given pseudoword, this will result in low entropy (*H* = 0). If the participants’ responses are diverse, entropy increases, resulting in a higher measure of *H*. For a given pseudoword *x*, item-level entropy (*H*) can be calculated with the following formula:

$$ H(x)=-{\sum}_{i=1}^n\left({p}_i\left(x={y}_i\right)\times {\log}_2\left({p}_i\left(x={y}_i\right)\right)\right) $$

The Python script used to calculate the entropy for our data set can be found here: https://osf.io/x63bf/. As we were interested in the diversity of vowel responses, we excluded trials where children made consonant errors.

Given our design, we were also able to calculate participant-level entropy: As the vowel grapheme was identical for all pseudowords, participant-level entropy reflects the extent to which a given participant pronounces the grapheme *a* in the same way across conditions. We explored item-level entropy to assess differences in entropy across grade, and participant-level entropy, to assess differences as a function of reading ability and item type.

##### Item-level entropy

**Fig. 1:**
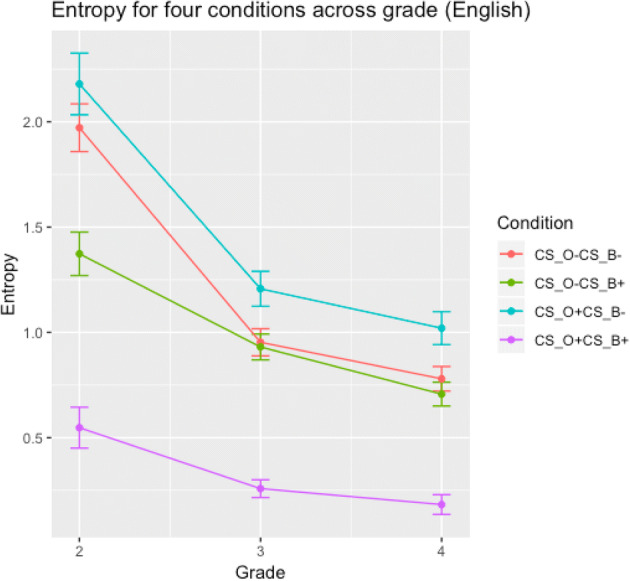
Decrease in entropy across grades for experiment 2. Error bars represent the standard error of the mean

The entropy for each item, both across the whole sample and split across grades, can be found here: https://osf.io/x63bf/, and the averages per condition are summarised in Table 4 and Fig. 1.

**Table 4:** Entropy values for the whole sample in experiment 1, and split by grade, across conditions. Mean (SD)

Condition	Whole sample	Grade 2	Grade 3	Grade 4
CS_GPC_O_+CS_GPC_B_+ (hact)	0.5 (0.3)	0.5 (0.4)	0.3 (0.2)	0.2 (0.2)
CS_GPC_O_-CS_GPC_B_+ (wact)	1.5 (0.4)	1.4 (0.4)	0.9 (0.3)	0.7 (0.2)
CS_GPC_O_+CS_GPC_B_- (kalt)	2.3 (0.6)	2.2 (0.6)	1.2 (0.4)	1.0 (0.3)
CS_GPC_O_-CS_GPC_B_- (wald)	1.9 (0.3)	2.0 (0.5)	1.0 (0.3)	0.8 (0.2)

The item entropies were submitted to a 4 × 3 mixed ANOVA in which Condition had 4 between-item levels and Grade had 3 within-item levels. Item number was treated as a within-participant factor across grades. We found a main effect of Condition, *F*(3,68) = 52.1, *p* < 0.0001, a main effect of Grade, *F*(3,140) = 266.1, *p* < 0.0001, and an interaction between Condition and Grade, *F*(3,140) = 14.9, *p* < 0.0001. Across grades, entropy decreased for all conditions (Fig. 1). A Bonferroni-corrected series of post-hoc *t* tests showed that entropy in grade 2 differed significantly from entropy in grades 3 and 4, *p* < 0.0001, while entropy in grades 3 and 4 did not differ significantly, *p* = 0.3. Across all grades, entropy was lowest for the CS_GPC_O_ + CS_GPC_B_+ (hact) condition: Entropy in this condition differed compared to all other conditions, *p* < 0.0001. There was also a significant difference in entropy between the CS_GPC_O_ + CS_GPC_B_- (kalt) and the CS_GPC_O_-CS_GPC_B_+ (wact) conditions, *p* < 0.0001; all other *ps* > 0.1. The significant interaction between condition and grade likely reflects the decrease in the difference between conditions across grades (Fig. 1).

##### Participant-level entropy

Participant-level entropy allows us to assess individual differences as a function of each child’s reading ability. We calculated the overall entropy in each participant’s vowel responses and the entropy for each participant for each of the four conditions. To explore the relationship between individual differences in reading ability and entropy, we generated a correlation matrix including overall entropy, the four entropy values for the separate conditions, TOWRE raw score, and TOWRE standard score. This gives us 15 *p* values; to adjust for multiple comparisons, we therefore used the significance threshold of 0.05/15 = 0.003 as the new alpha level. The correlation matrix is presented in Table 5. Critically, there was a significant negative correlation between entropy for the CS_GPC_O_+CS_GPC_B_+ (hact) condition and raw TOWRE reading ability, which reflects lower entropy (more consistent vowel pronunciations) for children with higher reading ability.

**Table 5:** Correlation matrix showing relationship between reading ability and entropy for the four different conditions in experiment 1

	Overall H	TOWRE Raw	TOWRE standard	CS_O_+CS_B_+	CS_O_+ CS_B_-	CS_O_-CS_B_+	CS_O_-CS_B_-
Overall H		− 0.22	− 0.06	0.46*	0.6*	0.67*	0.6*
TOWRE Raw	0.0851		0.81*	− 0.47*	− 0.21	− 0.16	− 0.26
TOWRE standard	0.6484	< 0.0001		− 0.3	− 0.11	− 0.13	− 0.13
CS_O_+CS_B_+	0.0002	0.0001	0.0175		0.19	0.42	0.18
CS_O_+CS_B_-	< 0.0001	0.1037	0.409	0.1431		0.25	0.37
CS_O_-CS_B_+	< 0.0001	0.233	0.3337	0.0007	0.0564		0.18
CS_O_-CS_B_-	< 0.0001	0.042	0.3237	0.1681	0.0033	0.1743	

The correlation coefficients are above the diagonal empty cells; the *p* values are below

*Significance after Bonferroni correction

### Discussion

By the end of grade 2, children learning to read in English already give some context-appropriate vowel responses when context-sensitive GPCs signal a change compared to the simple pronunciation. This suggests that children sometimes apply context-sensitive GPCs by the end of grade 2, even in the absence of their explicit instruction. In our sample, the pattern of vowel responses was stable across grades. This is in conflict with the results of a previous study looking at children’s pseudoword pronunciations (Treiman et al., 2006): here, the authors found an increase in sensitivity to context-sensitive GPCs up to fifth grade. This difference across studies may be driven by the pseudoword characteristics: It is possible that some context-sensitive GPCs are learned later than others, and ours may have an early age-of-acquisition.

The age-of-acquisition is likely to be driven by two factors: the frequency with which a given correspondence is encountered and its consistency. To assess whether there may be differences between our items and those of Treiman et al. (2006), we calculated the frequency and consistency of the orthographic clusters in question. Both our study and that of Treiman et al. (2006) included items with the *[w]a* and *[qu]a*, as well as the *a[l][C]* orthographic clusters. In addition, Treiman et al. (2006) included items with the bodies *-ead*, *-ild*, *-ind*, *-old*, *-olt*, and -*ook*. From a list of 6295 monosyllabic words, extracted from the English Lexicon Project (Balota et al., 2007), we calculated (1) how frequently each orthographic cluster occurs, (2) the consistency (i.e. the proportion of the most frequent pronunciation relative to the overall number of occurrences), and (3) the entropy associated with the possible pronunciations, which has the advantage over the consistency measure in the sense that it can account for more than two possible pronunciations (Siegelman et al. in press). These three measures are summarised in Table 6. The frequency was substantially higher for those items which occurred in both studies (average frequency = 43.6 occurrences), compared to those which occurred only in Treiman et al. (2006) (average frequency = 9.3 occurrences). The consistency was lower and entropy was higher for the letter clusters which occurred in both studies (0.63 and 0.43, respectively) compared to those that occurred in Treiman et al. (2006) only (0.81 and 0.15, respectively). Thus, it may be that the difference between the studies is due to children learning higher frequency regularities at younger ages, but further research would be needed to test this directly.

**Table 6:** Frequency, consistency, and entropy associated with the critical letter clusters of Treiman et al. (2006) and the current study

Orthography	Frequency	Consistency	Entropy	In study
alC	69	0.61	0.46	Treiman et al., Schmalz et al.
ead	14	0.57	0.3	Treiman et al.
ild	6	0.5	0.3	Treiman et al.
ind	9	0.89	0.15	Treiman et al.
old	10	1	0	Treiman et al.
olt	5	1	0	Treiman et al.
ook	12	0.92	0.12	Treiman et al.
qua	17	0.71	0.35	Treiman et al., Schmalz et al.
wa	45	0.56	0.48	Treiman et al., Schmalz et al.

In contrast to a similar study with adults (Schmalz et al., 2014), the optimisation procedure gave rather poor model fits. This result is likely caused by the relatively many unexpected responses of the children in our sample. This noisiness of the vowel responses might be systematic, reflecting the use of additional regularities (e.g. the final *e* in some pseudowords may have caused participants to pronounce the vowel as /æɪ/), or interference from the lexical route biasing the pronunciation to be similar to real-word orthographic neighbours. However, this was not confirmed by our analyses: relaxing the constraint that the weightings for our proposed GPCs add up to one did not improve the model fit. Alternatively, the connections between graphemes and phonemes might not yet be fully established in beginning readers, causing some random variability in children’s vowel responses. We tested for this possibility using the entropy measures and will follow up on it in experiment 3.

In a set of exploratory analyses, we calculated item- and participant-level entropy of the children’s vowel responses. We found an overall decrease in entropy across age, especially between grades 2 and 3. Even though the children are already able to apply context-sensitive GPCs, this knowledge appears to become more fine-tuned and less noisy over time. The participant-level analyses showed that the relationship between reading ability and entropy was especially strong in the CS_GPC_O_+CS_GPC_B_+ (hact) condition, where all cues favour the default /æ/ pronunciation. Thus, even in this simple condition, superior reading ability appears to be associated with a more consistency in the pronunciation to pseudowords with similar characteristics.

## Experiment 2: modelling vowel responses in German

The second experiment mirrors the methods and analyses of experiment 1, but with a sample of German-speaking children reading German pseudowords. For the optimisation procedure, we may expect better model fits: In English, there is a high degree of unpredictability in the pronunciation of graphemes (Borgwaldt et al., 2004; Schmalz, Marinus, Coltheart, & Castles, 2015; Seymour, Aro, & Erskine, 2003). This might be why we found a large number of unexpected vowel responses. German, in contrast, has a more transparent orthography. Most GPC inconsistencies, for monosyllabic words, are associated with vowel length (Ziegler, Perry, & Coltheart, 2000). German vowels can either have long or short pronunciations (e.g. *Staat*, /ʃta:t/, state; *Stadt*, /ʃtat/, city). There are words without explicit vowel length markings: *Blond* (/blɔnt/, blonde) and *Mond* (/mo:nt/, moon) have the same body, but different vowel lengths. There is a language-level regularity, however: when the vowel is followed by only one consonant, the vowel tends to be long (e.g. *Wal*, /va:l/, whale). When the vowel is followed by two or more consonants, the vowel tends to be short (e.g. *Wald*, /valt/, forest) (Perry, Ziegler, Braun, & Zorzi, 2010; Ziegler et al., 2000). These two CS_GPCs, *V[C][C]* ➔ short vowel and *V[C]* ➔ long vowel, have been termed *super-rules* (hereafter: SR), as they are valid for a whole class of graphemes (i.e. all vowels).

These features of the German orthography allow us to create pseudowords where CS_GPCs either support or contradict a simple GPC. Most instances of unmarked vowels in German are short (Perry, Ziegler, Braun, et al., 2010); therefore, we treat short vowel pronunciations as the simple GPC. When there is one consonant in the body (e.g. in the pseudoword *BLAF*), SRs contradicts the simple GPC by signalling a long vowel (SR-). When there are two or more consonants (e.g. *BAMT*), the SR supports the context-insensitive pronunciation (CSR+). We can also take into account the identity of the subsequent consonants (i.e. the body), which sometimes contradict the cues provided by the SR: While the body -*agd* has two consonants, all words containing this body have a long vowel pronunciation (e.g. *Jagd*, /ja:kt/, hunt). Thus, the pseudoword *BLAGD* is SR+CS_GPC_B_-. Conversely, all words with the body -*it* have a short vowel pronunciation (e.g. *mit*, /mɪt/, with), therefore the pseudoword *GIT* is SR-CS_GPC_B_+. The pseudoword *BAMT* is SR+CS_GPC_B_+, as all GPCs signal a short vowel, and the word *BLAF* is SR-CS_GPC_B_-, as the number of consonants and the CS_GPC_B_ (e.g. *Schaf*, /ʃa:f/, sheep) signal a long vowel. With these four conditions being roughly equivalent to experiment 1, we aim to assess whether the optimisation procedure yields better model fits for children learning to read in a more transparent orthography.

### Methods

#### Participants

The participants were 55 children from the Berlin-Brandenburg area, who had participated in a different study at Potsdam University and had agreed to return for another testing session. Nineteen were in grade 2, 19 in grade 3, and 17 in grade 4. One participant from grade 2 had missing values for reading ability: their data is excluded from the participant-level analyses. Participant details are described in Table 7. Reading ability was measured with the sight word reading test of the SLRT II (Moll & Landerl, 2010).

**Table 7:** Participant characteristics experiment 2: mean (SD)

Measure		Grade 2	Grade 3	Grade 4
Age (months)		96.8 (4.4)	108.9 (7.6)	120.1 (4.9)
SLRT raw score		38.2 (15.9)	62.8 (29.0)	79.1 (17.2)
SLRT percentile		62.6 (26.9)	66.3 (20.6)	60.8 (25.7)

#### Items and procedure

The items are listed in Appendix 2. The four conditions are as described above: SR+CS_GPC_B_+ (*BAMT*), SR-CS_GPC_B_+ (GIT), SR+CS_GPC_B_- (*BLAGD*), and SR-CS_GPC_B_- (*BLAF*). The items were presented on flashcards, each for an unlimited time, in a fixed random order. All pseudowords were spelled in capital letters: In German, the capitalisation of the first letter can serve as a cue to word class, which has been shown to affect pseudoword pronunciations (Campbell & Besner, 1981). The children’s responses were transcribed offline by a native German speaker: items with consonant or vowel errors^2^ were marked as incorrect and non-responses were noted; for correct responses, we scored whether the vowel had been pronounced as long or short.

### Results

The proportion of short and long vowel responses and errors are summarised in Table 8.

**Table 8:** Summary of responses across conditions in experiment 2; percentage of responses (SD)

Condition	Grade	Short vowel response	Long vowel response	Errors
SR+CS_B_+	2	69.3% (13.4)	19.8% (10.3)	10.9% (8.7)
	3	76.8% (14.4)	16.8% (13.2)	6.3% (5.9)
	4	73.3% (8.7)	17.3% (11.1)	9.0% (8.7)
SR-CS_B_+	2	65.0% (24.1)	22.0% (20.0)	13.0% (8.5)
	3	74.0% (19.4)	18.3% (18.3)	7.7% (7.4)
	4	69.9% (21.6)	23.2% (20.8)	6.9% (10.9)
SR+CS_B_-	2	71.3% (20.8)	17.4% (16.6)	11.3% (13.2)
	3	65.2% (21.0)	22.7% (19.1)	12.1% (15.5)
	4	60.2% (20.4)	33.9% (19.8)	5.9% (7.9)
SR-CS_B_-	2	46.1% (18.5)	43.2% (19.5)	10.7% (6.0)
	3	46.8% (24.3)	43.3% (24.6)	9.8% (10.5)
	4	40.2% (21.0)	51.4% (23.8)	8.4% (10.8)

Here we present the percentages of different responses rather than the total number, because the number of trials was not equal across the four conditions (see Appendix 2)

#### Using optimisation to quantify sensitivity to different types of GPCs

For the German data, there are only two plausible responses for a given item: Either a long or a short vowel response. Thus, the fitting of two sets of equations should predict the participants’ responses for each item:

$$ P(S)={\beta}_{\mathrm{Simple}\ \mathrm{GPC}}\times P\left(S|\mathrm{Simple}\ \mathrm{GPC}\right)+{\beta}_{\mathrm{CS}\mathrm{R}}\times P\left(S|\mathrm{SR}\right)+{\beta}_{{\mathrm{CS}}_{\mathrm{B}}}\times P\left(S|{\mathrm{CS}}_{\mathrm{B}}\right) $$

$$ P(L)={\beta}_{\mathrm{Simple}\ \mathrm{GPC}}\times P\left(L|\mathrm{Simple}\ \mathrm{GPC}\right)+{\beta}_{\mathrm{CS}\mathrm{R}}\times P\left(L|\mathrm{SR}\right)+{\beta}_{\mathrm{CS}\_\mathrm{B}}\times P\left(L|{\mathrm{CS}}_{\mathrm{B}}\right) $$

where *P*(*S*) and *P*(*L*) stand for the observed proportions of short and long vowel responses, respectively; the weights *β*_*j*_ are the weights for each GPC-type *j* at which the model arrives; and the second term in the product reflects the language-level probabilities of each type of response, given each GPC type. As in the English model, the weights were constrained by the criteria *β*_*j*_ ∈ [0, 1] and ∑*β*_*j*_ = 1. Table 9 shows the weights across grades.

**Table 9:** Averaged weights (SD) for the sensitivity to simple GPCs, SRs, and CS_B_s across grade in the German sample (experiment 2)

Predictor	Simple GPC	SR	CS_B_
Grade 2	0.60 (0.36)	0.34 (0.32)	0.06 (0.10)
Grade 3	0.62 (0.39)	0.27 (0.29)	0.11 (0.17)
Grade 4	0.51 (0.40)	0.29 (0.29)	0.19 (0.21)

The model fits across grades were 0.42, 0.49, and 0.46, respectively. Again, these fits are substantially lower than those we observed for German adults (0.84). Relaxing the ∑*β*_*j*_ = 1 constraint did not substantially change the weightings (grade 2: *β*_sGPC_ = 0.65, *β*_SR_ = − 0.17, and *β*_CS_B_ = 0.09; grade 3: *β*_sGPC_ = 0.64, *β*_SR_ = 0.18, and *β*_CS_B_ = 0.12; grade 4: *β*_sGPC_ = 0.53, *β*_SR_ = 0.20, and *β*_CS_B_ = 0.20) or the model fits (0.42, 0.49, and 0.46, across grades).

#### Item- and participant-level entropy

As for the English sample, we investigated whether German-speaking children showed variability in their pseudoword pronunciations, despite the relatively high transparency. Again, we calculated both item-level and participant-level entropy. Entropy was calculated based on correct responses only, meaning that there were only two possible responses: either long or short vowel pronunciation (see Footnote 2). The average item-level entropy values are summarised in Table 10.

**Table 10:** Entropy values for the whole sample in experiment 2, and split by grade, across conditions. Mean (SD)

Condition	Whole sample	Grade 2	Grade 3	Grade 4
SR+CS_B_+	0.6 (0.3)	0.7 (0.3)	0.4 (0.2)	0.3 (0.2)
SR-CS_B_+	0.7 (0.2)	0.7 (0.4)	0.4 (0.2)	0.4 (0.1)
SR+CS_B_-	0.8 (0.2)	0.6 (0.3)	0.5 (0.1)	0.4 (0.1)
SR-CS_B_-	0.9 (0.1)	0.9 (0.1)	0.5 (0.1)	0.4 (0.1)

##### Item-level entropy

**Fig. 2:**
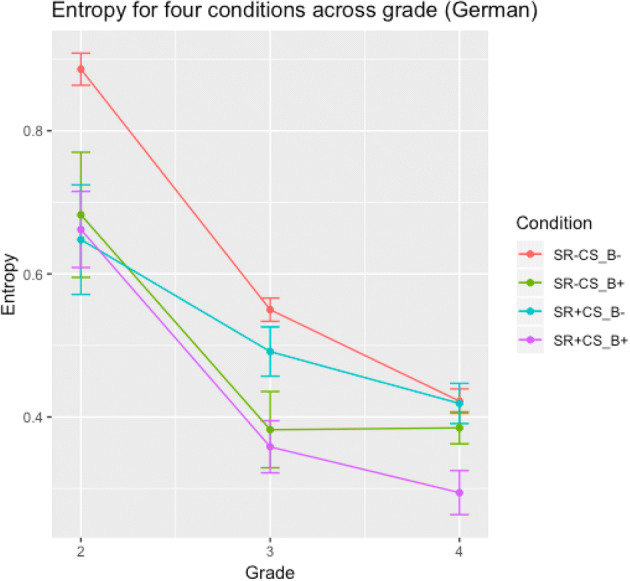
Decrease in entropy across grades in experiment 3. Error bars represent the standard error of the mean

Again, we performed a 4 × 3 ANOVA (Condition × Grade), with item number as a repeated factor across grades. We found a main effect of Condition, *F*(3,86) = 7.3, *p* = 0.0002, a main effect of Grade, *F*(3,176) = 254.0, *p* < 0.0001, and an interaction between the two, *F*(3,176) = 4.3, *p* = 0.0061. A series of Bonferroni-corrected *t* tests showed that entropy in grade 2 was significantly higher compared to grades 3 and 4 (*p* < 0.0001), while the difference between grades 3 and 4 was only marginal, *p* = 0.0530. In a series of Bonferroni-corrected *t* tests between the four conditions, the SR-CS_GPC_B_- (BLAF) condition differed significantly from the SR+CS_GPC_B_+ (BAMT) condition, *p* < 0.0001, and from the SR-CS_GPC_B_+ (GIT) condition, *p* = 0.0130, all other *p* > 0.2. The interactive pattern is illustrated in Fig. 2: despite the significant interaction, the graph shows that entropy decreased for all four conditions across grade.

##### Participant-level entropy

We generated a correlation matrix of the participant-level entropy overall, split by conditions, and raw and standardised reading scores. The correlation matrix is presented in Table 11; again, the alpha level was lowered to 0.003 to correct for multiple comparisons.

**Table 11:** Correlation matrix showing relationship between reading ability and entropy for the different conditions in experiment 2

	Overall H	SLRT Raw	SLRT percentile	SR+	SR+	SR-	SR-
				CS_B_+	CS_B_-	CS_B_+	CS_B_-
Overall H		− 0.11	− 0.22	0.31	0.42*	0.16	0.01
SLRT raw	0.42		0.68*	− 0.46*	− 0.36	− 0.1	− 0.33
SLRT percentile	0.1093	< 0.0001		− 0.48*	− 0.38	− 0.17	− 0.18
SR+	0.0219	0.0004	0.0002		0.31	0.35	0.24
CS_B_+							
SR+	0.0016	0.0071	0.0046	0.0205		0.03	0.15
CS_B_-							
SR-CS_B_+	0.2387	0.4773	0.2067	0.0085	0.8381		0.17
SR-CS_B_-	0.917	0.0136	0.1961	0.0748	0.2873	0.2061	

The correlation coefficients are above the diagonal empty cells; the *p* values are below

*Significance after Bonferroni correction

Raw reading ability was positively correlated with the reading percentile. Importantly, both raw reading score and percentile on the SLRT was correlated with entropy in the SR+CS_GPC_B_+ (BAMT) condition, with better readers (both in absolute terms and relative to their grade level) showing smaller entropy.

### Discussion

The results of experiment 2 are broadly in line with those of experiment 1. Using optimisation to select a set of weights to quantify the degree to which children rely on different types of GPCs gave poor model fits. Thus, vowel responses of children, even when they are learning to read in a transparent orthography, appear to be too unstable to make this approach viable. In terms of entropy, we replicated a correlation between reading ability and entropy in the SR+CS_GPC_B_+ (BAMT) condition. Thus, in both English and German, better readers are more consistent in their pronunciations in an unambiguous context than poor readers.

## Experiment 3: test-retest reliability

In the first two experiments, we showed that there was within-participant variability in children’s vowel pronunciations. The pronunciations may therefore be affected, to some extent, by a random error term, which would affect the final pronunciation after a set of plausible pronunciations have been pre-activated. This does not follow unambiguously from the previous experiments, however: As the grapheme *a* was presented in different contexts, it is possible that different children relied on different regularities, which may reflect both their reading experience and their ability to pick up on subtle regularities. If the variability indeed reflects random noise rather than unmeasured systematic factors, we should expect that children will not be consistent in their pronunciation of the same pseudoword across testing sessions. In experiment 3, we aimed to explore whether there is such within-participant variability for repeated items.

### Methods

#### Participants

The participants were children attending two different schools in rural New South Wales (Australia). Of the children included in the current analyses, two were from grade 1, three from grade 2, five from grade 3, and 7 from grade 4. These 17 children were selected from a larger pool of participants (described in detail in Schmalz, 2015), because they had completed two experimental sessions, which were at least 24 h apart. In each of these two sessions, the children were presented with the same experimental pseudowords (described below), meaning that each child read aloud each pseudoword twice. The participant’s ages and reading ability, as measured by the TOWRE sight word reading test (Torgesen et al., 1999) and TOWRE *z*-scores according to Australian norms (Marinus, Kohnen, & McArthur, 2013), are summarised in Table 12.

**Table 12:** Participant characteristics of the sample in experiment 3: mean (SD)

	Grade 1	Grade 2	Grade 3	Grade 4
Age (months)	86.0 (0.0)	101.0 (3.6)	114.2 (2.8)	121.7 (3.7)^1^
TOWRE raw score	63.5 (14.8)	59.7 (13.8)	67.4 (5.3)	73.0 (12.5)
TOWRE *z*-score	1.6 (1.4)	0.7 (1.3)	0.3 (0.9)	0.6 (1.5)

^1^There was one missing value for age; the average and SD for this cell are calculated based on the remaining 6 participants

#### Items and procedure

The items were the same as in experiment 1, though one item was replaced (see Appendix 1). The transcriptions of children’s vowel responses can be found on the OSF site linked above.

The children were tested individually in a quiet room at their school. For the experimental task, they were presented with each pseudoword, printed on a flashcard, in a fixed random order, and were given an unlimited amount of time to read each item aloud. Their responses were audio-recorded. In addition to the experimental pseudowords, the set of items also contained 20 filler pseudowords, which were randomly intermixed with the experimental pseudowords. These were different across the two sessions: In one session, the filler pseudowords had bodies which occur in many real words (high-frequency bodies), and in the other session, the filler pseudowords had bodies which occur in few real words (low-frequency bodies). The type of filler words was counterbalanced across session order: the original aim of the study was to see if introducing pseudowords with either high- or low-frequency bodies would increase the reliance on larger sublexical units (cf. Goswami, Ziegler, Dalton, & Schneider, 2003).^3^

### Results

For the experimental pseudowords, the children’s vowel responses were transcribed by a trained phonologist, who was unaware of the experiment’s aim. Items with consonant errors or non-responses were scored as incorrect. The transcribed data can be found here: https://osf.io/qnuc2/. The number of different types of responses, across conditions, is summarised in Table 13.

**Table 13:** Summary of responses across conditions in experiment 3; average number of responses (SD)

Condition	Filler type	/æ/	/ɔ/	/o:/	Consonant error/non-response	Other vowel response
CS_O_+CS_B_+	High body frequency	13.6 (1.7)	0.2 (0.4)	0 (0)	1.3 (1.4)	2.9 (1.0)
	Low body frequency	13.9 (1.4)	0.1 (0.2)	0 (0)	0.9 (1.5)	3.1 (1.1)
CS_O_-CS_B_+	High body frequency	10.5 (2.8)	3.7 (2.8)	0.5 (0.6)	2.0 (2.3)	1.4 (1.4)
	Low body frequency	11.1 (2.4)	3.6 (2.9)	0.4 (0.6)	1.9 (2.1)	1.0 (1.1)
CS_O_+CS_B_-	High body frequency	6.0 (3.3)	4.2 (4.5)	1.5 (1.8)	2.1 (2.1)	4.1 (2.5)
	Low body frequency	6.3 (3.9)	4.2 (4.6)	2.1 (2.9)	1.8 (2.1)	3.6 (2.5)
CS_O_-CS_B_-	High body frequency	1.6 (2.5)	10.1 (5.5)	3.4 (2.5)	1.6 (1.7)	1.2 (1.9)
	Low body frequency	2.2 (3.0)	8.0 (4.7)	3.5 (3.9)	1.2 (1.6)	3.1 (1.9)

The test-retest reliability of children’s responses was calculated based on all responses (i.e. including incorrect responses). For each child, if their response to a given pseudoword contained the same vowel response or status as incorrect, it was coded as 1. If the vowel response was different across sessions, or the response was scored as incorrect in one session but not the other, it was coded as 0. This allowed us to calculate the degree of overlap between the pronunciations across the two sessions for each child. On average, the proportion of overlap across sessions was 0.59 (SD = 0.10), ranging from 0.47 to 0.79.

Across grades, the proportion of overlap was 0.59 (SD = 0.13) for grade 1, 0.60 (SD = 0.10) for grade 2, 0.57 (SD = 0.06) for grade 3, and 0.60 (SD = 0.12) for grade 4. An ANOVA with grade as a four-level independent variable and participant-level proportion of overlap as the dependent variable showed no main effect of grade, *F*(1,15) < 0.1, *p* > 0.9. A correlation analysis showed that the degree of overlap was positively, but not significantly correlated with reading ability: with raw TOWRE scores, *r*(16) = 0.31, *p* = 0.2, and with standardised scores, *r*(16) = 0.38, *p* = 0.1.

### Discussion

To our knowledge, this study is the first to assess the reliability of pseudoword responses in any population. The overlap in the pronunciations was rather low: In about 40% of the cases, the children gave a different pronunciation in the first compared to the second session. The sample is too small to draw conclusions about the presence or absence of correlations with child-level factors (an observed correlation would need to be greater than approximately 0.5 to reach the significance threshold of *p* < 0.05 with a sample of 17). There was a tendency, however, for better readers to give more consistent responses.

To some extent, the different filler pseudowords which were used across sessions may have affected the results (see Footnote 3), by biasing the children towards relying on CS_GPC_B_s when the filler pseudowords had high-frequency bodies and towards simple GPCs when the fillers had low-frequency bodies. Furthermore, the pseudowords used in the current study were difficult, as they contained CS_GPCs, consonant clusters, and had low similarity to existing words; the difficulty of these pseudowords may have increased the variability compared to pseudowords which are more word-like and thus more representative of the written items that children encounter during reading. However, even if the low consistency across sessions is driven or affected by the filler pseudowords or item-level factors, the results suggest that the responses which children give are relatively unstable across different situations. Thus, models of single-word reading in children should work towards being able to simulate within-subject variability in pseudoword reading responses.

## General discussion

Across three experiments in children learning to read, and across two languages, we showed that there is variability in vowel pronunciations. To a large extent, this variability appears to be unsystematic, as the same children pronounced the same graphemes or even whole pseudowords differently at different times. We further showed that a mathematical modelling approach to infer the extent to which the reader relies on different types of GPCs yields poor model fits for children, even though we have previously successfully applied this approach to data from adults. The unsystematic variability in vowel pronunciations is a likely cause of the poor model fits for children.

We explored the variability of vowel pronunciation by introducing an entropy measure. Using this measure, we found that pronunciation variability was lower for better than for poorer readers. In both experiments 1 and 2, the correlation was strong and significant for the unambiguous pronunciation. These findings are in line with the results of an earlier study which assessed intra-individual variability in children’s reading aloud reaction times (Marinus & de Jong, 2010). Here, the difference between children with dyslexia and the control group was largest in the simplest condition: namely, reading aloud of three-letter words. It is possible that the responses to more difficult items are affected to a greater extent by cognitive skills beyond GPC knowledge, such as blending ability or verbal short-term memory, which is likely to increase the variability for all participants, regardless of their reading ability (Pritchard et al., 2012).

The entropy measure could be further explored by future research. One question of interest is whether children with dyslexia would show higher entropy values, when asked to respond to the same pseudowords or graphemes across situations. Children with dyslexia are often impaired in pseudoword reading (Rack, Snowling, & Olson, 1992). In line with the currently reported results, this consistent finding could be interpreted as resulting from unstable representations of the letter-phoneme correspondences. Thus, a future study could assess whether entropy differs in children with dyslexia compared to a control group, as well as assessing the relationship between an individual’s pseudoword reading aloud entropy and reading ability in a more controlled study.

The finding that pseudoword responses are variable, even within participants, is relevant to computational models of reading, as the currently implemented models do not predict that the same individuals may give different pronunciations to the same pseudowords. The Dual Route Cascaded model assumes that pseudoword pronunciations occur as a result of the application of GPC rules (Coltheart et al., 2001). Different types of rules can be implemented to reflect differences *across* individuals: a given individual may know the context-sensitive rule *[w]a* ➔/ɔ/, and accordingly pronounce words with this orthographic pattern with the vowel /ɔ/, while another individual may not know this rule and pronounce the vowel as /æ/. A different class of computational models, based on connectionist networks, assumes that knowledge of sublexical print-to-speech correspondences develops through experience with real words (Perry et al., 2007; Perry et al., 2010; Plaut et al., 1996; Seidenberg & McClelland, 1989). During the learning process, the system extracts regularities about CS_GPCs. In these models, the end state behaviour is affected by system and input characteristics (Patterson, Seidenberg, & McClelland, 1989; Plaut et al., 1996). By changing model parameters or the training material, again, these types of models are able to simulate differences across but not within participants.

To simulate the lack of consistency of the responses *within* individual children, one could add noise to the system. Such a model could first activate a set of plausible candidate pronunciations (e.g. for the grapheme *a*, the phonemes /æ/, /ɔ/, /o:/, /æɪ/, /ɐ/), and the final output could be based on the context of the grapheme, as well as a random error term. Future research is needed to determine, on the behavioural level, whether there are participant-level characteristics, beyond reading skill, which determine the extent to which random noise affects a given participant’s pseudoword responses.

## Appendix 1. Items used in experiments 1 and 3

Condition CS_GPC_O_+CS_GPC_B_+

hact, hangst, kadge, kamb, kazz, phamb, phangst, sangst, slact, slazz, slact, slangst*, stramb, tapse, tazz, tradge, trapse, trazz

Condition CS_GPC_O_-CS_GPC_B_+

quadge, quamb, quangst, quapse, quazz, squazz, swact, swamb, swangst, swazz,

twact, twadge, twangst, twapse, twazz, wadge, wamb, wapse

Condition CS_GPC_O_+CS_GPC_B_-

clatt, halse, kalk, kalse, kalt, phald, phalk, phaltz, slaltz, stract**, strald, stralk, stralse, straltz, taltz, trald, tralse, tralt, tratt

Condition CS_GPC_O_-CS_GPC_B_-

qualk, qualse, qualtz, quatt, squald, squalk, squalse, squaltz, swaltz, twald, twalk, twalse, twalt, twaltz, wald, walse, walt, whald

*Presented in experiment 1 but not experiment 3

**Presented in experiment 3 but not experiment 1

## Appendix 2. Items used in experiment 2

Condition SR+CS_GPC_B_+

bamt, birt, blaft, bling, boft, brals, chrolf, falb, flarg, flerk, gärm, ginn, gralb, gunt, kall, kaxt, kerv, kluns, knell, pals, peld, pfern, pulk, purf, schern, spalf, stelf, sturg, zeng, zwurt

Condition SR-CS_GPC_B_+

bax, blex, blig, bres, flim, flis, git, glef, glip, krex, krin, krip, pfis, spic, stef, zwix, zwok

Condition SR+CS_GPC_B_-

bags, blags, füst, gleks, kagd, kagt, kets, pagt, pard, peks, poks, schagd, stard

Condition SR-CS_GPC_B_-

blaf, blen, blod, breg, brel, brul, flom, flüb, fryp, grät, grem, grom, grul, klid, klur, knul, krel, kril, krön, pflyp, pid, plät, plön, prod, schmün, schraf, schwüb, speg, zwül, zwun

## Footnotes

1 The phonetic transcriptions for English throughout this paper are based on Australian English (Cox & Palethorpe, 2007).

2 Note that in German, as opposed to English, vowel pronunciation, aside from length, is unambiguous. If a child, for example, pronounced a pseudoword with the grapheme *o* as /u:/, this was scored as an error, because words containing this grapheme can only have either an /ɔ/ or an /o:/ pronunciation.

3 Although this is not the focus of the current analyses, we assessed whether the presence of high-frequency bodies shifted the children’s responses away from relying on simple rules and towards relying on CS_GPCBs. When we looked at the number of regular responses (coded as 1) as opposed to any other response (coded as 0) as the dependent variable and filler type as the predictor variable, a generalised linear mixed effect model showed a marginally significant difference between low- and high-frequency filler types, *t* = 1.8250, *p* = 0.068, reflecting a slightly higher percentage of regular responses for the low body-N filler condition (48.1%) compared to the high body-N filler condition (45.3%).
